# Efficient generation of mouse models of human diseases via ABE- and BE-mediated base editing

**DOI:** 10.1038/s41467-018-04768-7

**Published:** 2018-06-14

**Authors:** Zhen Liu, Zongyang Lu, Guang Yang, Shisheng Huang, Guanglei Li, Songjie Feng, Yajing Liu, Jianan Li, Wenxia Yu, Yu Zhang, Jia Chen, Qiang Sun, Xingxu Huang

**Affiliations:** 1 Institute of Neuroscience, Chinese Academy of Sciences (CAS) Key Laboratory of Primate Neurobiology, CAS Center for Excellence in Brain Science and Intelligence Technology, Shanghai Institutes for Biological Sciences, CAS, 200031 Shanghai, China; 2 School of Life Science and Technology, ShanghaiTech University, 100 Haike Road, Pudong New Area, 201210 Shanghai, China; 3 CAS Center for Excellence in Molecular Cell Science, Shanghai Institute of Biochemistry and Cell Biology, Chinese Academy of Sciences, University of Chinese Academy of Sciences, 320 Yueyang Road, 200031 Shanghai, China

## Abstract

A recently developed adenine base editor (ABE) efficiently converts A to G and is potentially useful for clinical applications. However, its precision and efficiency in vivo remains to be addressed. Here we achieve A-to-G conversion in vivo at frequencies up to 100% by microinjection of ABE mRNA together with sgRNAs. We then generate mouse models harboring clinically relevant mutations at *Ar* and *Hoxd13*, which recapitulates respective clinical defects. Furthermore, we achieve both C-to-T and A-to-G base editing by using a combination of ABE and SaBE3, thus creating mouse model harboring multiple mutations. We also demonstrate the specificity of ABE by deep sequencing and whole-genome sequencing (WGS). Taken together, ABE is highly efficient and precise in vivo, making it feasible to model and potentially cure relevant genetic diseases.

## Abstract (web summary)

CRISPR-based base editors allow for single nucleotide genome editing in a range of organisms. Here the authors demonstrate the in vivo generation of mouse models carrying clinically relevant mutations using C→T and A→G editors.

## Introduction

Base editor (BE) systems convert C-to-T in vitro and in vivo^1–4^. However, 48% of genetic diseases involve C-to-T substitution, and modeling or correcting such diseases requires the adenine base editor (ABE) systems that convert A-to-G^5^. One ABE (version 7.10) has been recently developed by protein artificial evolution, which consists of a wild-type (ecTadA) and an engineered (ecTadA*) adenine deaminase domains fused in tandem to the N terminus of Cas9 D10A nickase^6^. Its success in A-to-G conversion in vitro prompted us to determine its efficacy in vivo. To this end, we targeted androgen receptor (*Ar*) and homeobox D13 (*Hoxd13*), which are known to be associated with androgen insensitivity syndrome (AIS) and Syndactyly, respectively^7–9^ (http://www.uniprot.org/docs/humsavar). We first generated mouse models carrying single novel clinical mutation of *Ar* and *Hoxd13* by microinjection of ABE. We also tested whether SpABE could be used in conjunction with SaBE3 to create complex mutations.

## Results

### Screening for efficient single guide RNAs (sgRNAs) in cultured N2a cells

**Fig. 1:**
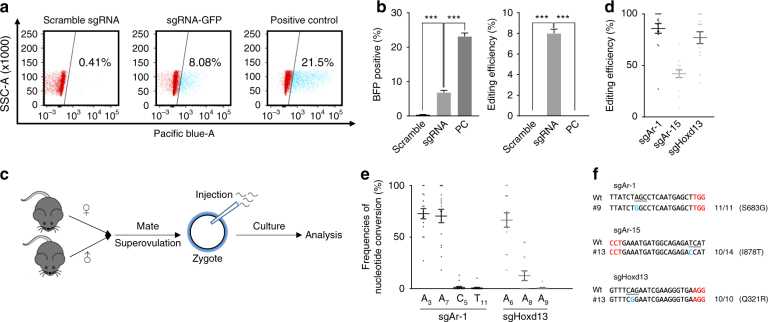
ABE-mediated efficient A-to-G conversion at *Ar* and *Hoxd13* loci in mouse embryos. **a** GFP-to-BFP conversion as a reporter for ABE-mediated base editing. **b** Analysis of base editing by FACS (left) and Sanger sequencing (right). Scramble: Scrambled sgRNA as negative control; sgRNA: sgRNA targeting GFP; PC: BFP expression plasmid only as positive control. Data were analyzed by Student’s *t*-test (***p < 0.001) and shown as mean ± s.e.m. (*n* = 3 from three independent experiments). **c** ABE-mediated base editing in vivo. ABE mRNA and sgRNA were co-injected into one-cell embryos, and the editing efficiency examined at blastocyst stage. **d** Efficiency of A-to-G substitution in mouse embryos. TA clones of PCR amplicons from the target regions in *Ar* (for sgAr-1, sgAr-15) and *Hoxd13* (sgHoxd13) were analyzed by Sanger sequencing. Each dot indicates one individual mouse. At least 10 TA clones were analyzed for each sample. **e** The editing frequencies of individual A-to-G conversion of samples described in **c** were analyzed. A_3_, A_7_, C_5_, and T_11_ indicate edited positions of the protospacer for sgAr-1; A_6_, A_8_, and A_9_ indicate edited positions of the protospacer for sgHoxd13. **f** Representative alignments of modified sequences from embryos after microinjection of ABE mRNA and sgRNAs into one-cell embryos. The PAM sequences and substitutions are highlighted in red and blue, respectively; the target codons are underlined; N/N represents positive colonies out of the total sequenced

We first tested whether ABE could function in mouse-derived Neuro-2a (N2a) cells, using a green fluorescent protein to blue fluorescent protein (GFP-to-BFP) conversion reporter system. Plasmids expressing sgRNA and ABE were co-transfected with the GFP-to-BFP reporter and the green-to-blue fluorescence conversion quantified by fluorescence-activated cell sorting (FACS). In all, 8% of BFP-positive cells were detected (Fig. 1a, b), and Sanger sequencing of the targeted region revealed A-to-G substitution at the same frequency (Fig. 1a, b), indicating that ABE is functional. We then screened 18 sgRNAs each for *Ar* and *Hoxd13* in the N2a cells and found 16 to be active (Supplementary Fig. 1c). Among them, three sgRNAs introduced mutations reproducing that seen in patients (http://www.uniprot.org/docs/humsavar): sgAr-1 and sgAr-15 caused S683G and I878T at the *Ar* locus linked to AIS, while sgHoxd13 caused Q321R at the *Hoxd13* locus associated with Syndactyly diseases (Supplementary Fig. 1a, b). The S683G and I878T mutations correspond to human homologous mutations S704G and I899T, respectively, which are seen in AIS patients characterized by sex reversal^8,10,11^ (Supplementary Fig. 1b), whereas the Q321R corresponding to Q306R are detected in Syndactyly patients^12,13^ (Supplementary Fig. 1b). Therefore, the three sgRNAs were selected for in vivo study.

### ABE-mediated efficient A-to-G substitutions in mouse embryos

We then attempted to edit the mouse embryos. ABE mRNA was co-injected with different sgRNAs into one-cell embryos (Fig. 1c), most of which developed normally to blastocyst (17 out of 18, 20 out of 22, and 13 out of 14 for sgAr-1, sgAr-15, and sgHoxd13, respectively) (Supplementary Fig. 2e), indicating that ABE did not affect embryo development. Analysis of 14–16 blastocysts revealed highly efficient editing, with the expected substitutions detected in all embryos and the mutation frequencies ranging from 8 to 100% (Fig. 1e, Supplementary Fig. 2a–c). Then 16 embryos from both sgAr-1 and sgAr-15, and all 14 embryos from sgHoxd13 were used for genotyping.

Specifically, for sgAr-1, A-to-G substitutions occurred at positions 3 and 7 as expected (Fig. 1e, Supplementary Fig. 2a). As the substitution at position 3 is a silent mutation, only S683G mutation derived from A_7_ was detected in all embryos (Supplementary Fig. 2a, d). Besides, in Embryos #7, #12, and #16, unwanted C-to-G or C-to-A at position 5 and T-to-C at position 11 were observed at frequencies of 13, 8, and 10%, respectively (Supplementary Fig. 2a), leading to corresponding amino-acid changes (S682C, S682Y, and L684P, respectively) (Supplementary Fig. 2d). For sgAr-15, all embryos harbored A-to-G substitution only at position 4 at frequencies ranging from 8 to 71% (Fig. 1d, Supplementary Fig. 2b). Consequently, I878T amino-acid conversion occurred over all embryos (Supplementary Fig. 2b, d). Similarly, expected A-to-G substitution at position 6 of sgHoxd13 target site was detected in all the tested embryos (Fig. 1e, Supplementary Fig. 2c). Also, A-to-G substitution in the editing windows was detected at positions 8 (Embryos #1, 2, 5, 6, 9, 14) and 9 (Embryo #9) (Fig. 1e, Supplementary Fig. 2c). Consequently, expected amino-acid conversion Q321R occurred at high frequency (66.6%) compared with N322D (12.6%, the average frequency of the tested embryos) and N322S (0.9%, the average frequency of the tested embryos) (Fig. 1e, Supplementary Fig. 2c). Taken together, these results demonstrated that ABE efficiently edits endogenous genes in mouse embryos without impairing development. Of note, there is only one A in the editing window (protospacer positions 4–7) of sgAr-15, and so only one modified genotype was generated (Supplementary Fig. 2b, d), suggesting that it is desirable to select a sgRNA with one targetable site inside the edit window to achieve isogenic modification, which should produce isogenic homozygous mutation, a desirable outcome.

### Generation of pathogenic A-to-G mouse models

**Fig. 2:**
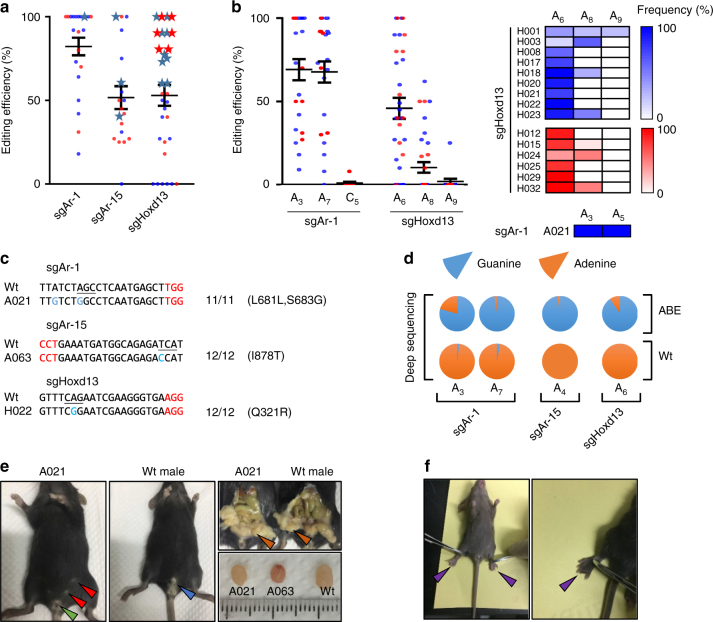
The generation of *Ar* and *Hoxd13* mutant mouse models by ABE. **a** The editing efficiencies were detected by Sanger sequencing of TA clones of mouse pups for sgAr-1, sgAr-15, and Hoxd13, respectively. Each dot indicates one individual mouse. At least ten TA clones were analyzed for each sample. Blue dots represent male mice and red dots represent female mice. Asterisks represent the mice with defects. **b** The editing efficiencies of different positions from individual samples described in **a** were analyzed. Left: A_3_, A_7_, and C_5_ indicate the edited positions of the protospacer for sgAr-1; A_6_, A_8_, and A_9_ indicate edited positions of the protospacer for sgHoxd13. Blue dots represent male mice and red dots represent female mice. Right: Editing frequencies in As of each mice with defects for sgHoxd13 and sgAr-1 are displayed in heatmap. Blue rectangles represent male mice and red rectangles represent female. Editing frequencies increase with the increase of color shades. **c** Representative alignments of modified sequences from pups after microinjection of ABE mRNA and sgRNAs into one-cell embryos. The PAM sequences and substitutions are highlighted in red and blue, respectively; the target codons are underlined; N/N represents positive colonies out of the total sequenced. **d** Characterization of the targeted modifications by deep sequencing. The frequencies were calculated from three (*n* = 3) replicates. **e** Sex reversal in founder mouse. Left: a 5-week-old mouse with female genitalia (green arrowhead) and nipples (red arrowheads); middle: Wt male with normal male genital (blue arrowhead); right: founder A021 with internal genitalia of male (orange arrowhead) and smaller testes and founder A063 with smaller testes. **f** Syndactyly phenotypes of founder mouse. Digits of mutant mouse are fused (purple arrowhead)

With this success, we set out to generate A-to-G mutant mice by co-injecting ABE mRNA (100 ng/µl) with sgRNA (50 ng/µl) into zygotes. A total of 80, 80, and 63 zygotes for sgAr-1, sgAr-15, and sgHoxd13 were injected and transferred into 4, 4, and 3 surrogate mice, which generated 22, 19, and 32 offspring, respectively (Table 1). Targeted mutations by sgAr-1, sgAr-15, and sgHoxd13 were detected in 100, 95, and 81% pups, respectively (Table 1, Supplementary Fig. 3a–d). For *Ar*, the editing frequencies ranged from 18 to 100% for sgAr-1 and from 25 to 100% for sgAr-15 (Fig. 2a, Supplementary Fig. 3a, b). As expected, sgAr-1 introduced mutation at position 3 (A_3_, L681L) and position 7 (A_7_, S683G) of the protospacer, while sgAr-15 introduced mutation at position 4 (A_4_, I878T) of the protospacer (Fig. 2b and Supplementary Fig. 3d). Also, unwanted C-to-T mutation was detected (Founder A020 from sgAr-1, at the frequency of 8%) (Fig. 2b, Supplementary Fig. 3a, d).

To determine whether the mutant mice displayed similar phenotype (AIS), we first confirmed the gender of the founders by genotyping of *Sry* gene (Y chromosome-specific gene) and found 15 out of the 22 for sgAr-1 (Founders A001-A014, A021), and 10 out of the 19 for sgAr-15 (Founders A046-A052, A061-A063) were male (Supplementary Fig. 4a). Interestingly, 1 out of the 15 male founders for sgAr-1 (Founder A021) and 3 out of the 10 founders for sgAr-15 (Founders, A061-A063) displayed typical AIS symptom with female external genitalia (Supplementary Fig. 4a). Founder A021 for sgAr-1 and A063 for sgAr-15 were selected to confirm the sex reversal phenotype by autopsy. As expected, both founders displayed smaller testes (Fig. 2e, Supplementary Fig. 4b). Then genomic DNA was isolated from four different tissues (heart, kidney, intestines, and testis) of Founder A021 and A063, and the region targeted by sgAr-1 and sgAr-15 was sequenced, which revealed 100% substitutions at two positions (A_3_, L681L and A_7_, S683G) for Founder A021 and a single position (A_4_, I878T) for Founder A063 (Supplementary Fig. 4d, e).

**Table 1:** Summary of the manipulation and genotyping of newborn pups

						Mutant ratio (%)	
Target gene	Methods		No. of examined embryos	No. of transferred embryos	No. of offspring (%)	No. of mutants/total offspring	No. of indels mutants/total mutants
*Ar*	sgAr-1+ABE mRNA	Microinjection	80	80	22 (28)^b^	22/22 (100)	0/22 (0)
*Ar*	sgAr-15+ABE mRNA	Microinjection	80	80	19 (24)^b^	18/19 (95)	0/18 (0)
*Hoxd13*	sgHoxd13+ABE mRNA	Microinjection	63^a^	60	32 (53)^b^	26/32 (81)	0/26 (0)

^a^Three embryos died during the microinjection

^b^Calculated from the number of examined embryos

Strangely, 1 male (Founder A014) harboring 100% substitution at position A_7_ for sgAr-1 (Supplementary Fig. 4a), and 1 male (A052) with 100% base substitution frequencies at position A_4_ for sgAr-15 (Supplementary Fig. 3a) did not display AIS phenotype. We hypothesized that mosaicism of the base editing caused the inconsistency of the phenotype. To test this, we assessed the editing efficiency of the testes from six mice (A001, A009, A014, A021, A052 and A063) harboring ~100% editing frequency in tails. Indeed, for A001, A009, A014, and A052, which did not display any phenotype, the frequencies of A-to-G substitutions at position A_7_ (S683G) in testes were only 42, 4, 17 and 38%, respectively. In contrast, for A021, A061, A062, and A063, which had sex reversal, the frequencies were 100, 69, 71 and 100%, respectively (Supplementary Fig. 4f, g). These data support our hypothesis that mosaicism in base editing in the testes underlies the variation in phenotype. To exclude any possible phenotype-associated non-target mutations beyond the editing window, we sequenced 500 bp target-flanking genomic region for all of the mice. No other point mutation was observed among the founders with AIS.

Regarding sgHoxd13, mutations at A_6_ (Q321R) and A_8_ (N322D) of the protospacer inside the editing window were detected at the efficiency from 17 to 100% (Fig. 2a, b and Supplementary Fig. 2c, d). Q321R mutation indeed caused fused digits in 15 (six females and nine males) out of the 32 (47%) mice (Fig. 2f), with all the founders with high efficiency of base editing in tails displaying the defects (Fig. 2a, b and Supplementary Fig. 2c, d). Genotyping of four tissues (heart, kidney, intestines, and ovary) from three digit-fused mice revealed a single Q321R mutation in one mouse and a compound (Q321R plus N322D) mutations in all tissues of the two other mice (Supplementary Fig. 4c). To our knowledge, this is the first time these mutations at *Ar* and *Hoxd13* loci in mice are shown to recapitulate the clinical defects in AIS and Syndactyly diseases.

To further assess the on-target and off-target effects, nine edited mice (A001, A004, and A015 for sgAr-1, A047, A052, and A063 for sgAr-15 and H008, H012, and H029 for sgHoxd13) were subjected to high-throughput deep sequencing. A total of ~100,000–3,400,000 reads for each sample were analyzed. The average conversions on targeted As were 79.3% (A_3_) and 97.7% (A_7_) for sgAr-1, 90.7% (A_4_) sgAr-15, and 81.3% (A_6_) for sgHoxd13 (Fig. 2d). Consistently, only one modification was induced by sgAr-15, further confirming the potential of ABE in generating isogenic mutation. Meanwhile, no indel was detected (Supplementary Fig. 7e), indicating that ABE is more precise than BE3 in vivo.

### Generation of mice by SpABE in combination with SaBE3

In the clinic, different kinds of mutations (including A to G and C to T) usually co-exist in the same patient^14,15^. We therefore attempted to introduce A-to-G and C-to-T substitutions simultaneously. To avoid possible competition between the BEs, we utilized SaBE3, which recognizes the PAM–NNGRRT, to target tyrosine receptor (*Tyr*) gene, and SpABE, which recognizes the NGG PAM, to target *Hoxd13*^16,17^; *Tyr* mutation is known to turn black hair to white hair^16–19^. Preliminary experiment in 16 one-cell embryos indicated that SaBE3 achieved 7–100% editing frequencies, with half the samples harboring indels at the frequencies from 7 to 84% (Supplementary Fig. 5a), while the other half carrying Q68stop often together with other types (S65F, P70S, P70T and P70L) of conversion, demonstrating that SaBE3-mediated base editing efficiently in vivo (Supplementary Fig. 5a).

We then repeated the above experiment, except that SpABE was used together with SaBE3. SaBE3 and SpABE mRNAs (total 100 ng/µl) were co-injected into one-cell embryos with *Tyr* and *Hoxd13* sgRNAs (total 50 ng/µl), and 15 blastocysts were collected and analyzed (Supplementary Fig. 5c). The results revealed that 13 carried expected base substitution at positon 6 (Q312R) at *Hoxd13*, 5 of which carried A-to-G substitution at position 8, generating unwanted N322D conversion (Supplementary Fig. 5b, c). At the *Tyr* locus, only 1 out of the 15 blastocysts harbored Q68stop conversion at 100% frequency, while 6 out of the 15 (40%) blastocysts harbored indels (Supplementary Fig. 5b, c). These results demonstrated simultaneous A-to-G and C-to-T substitutions.

**Fig. 3:**
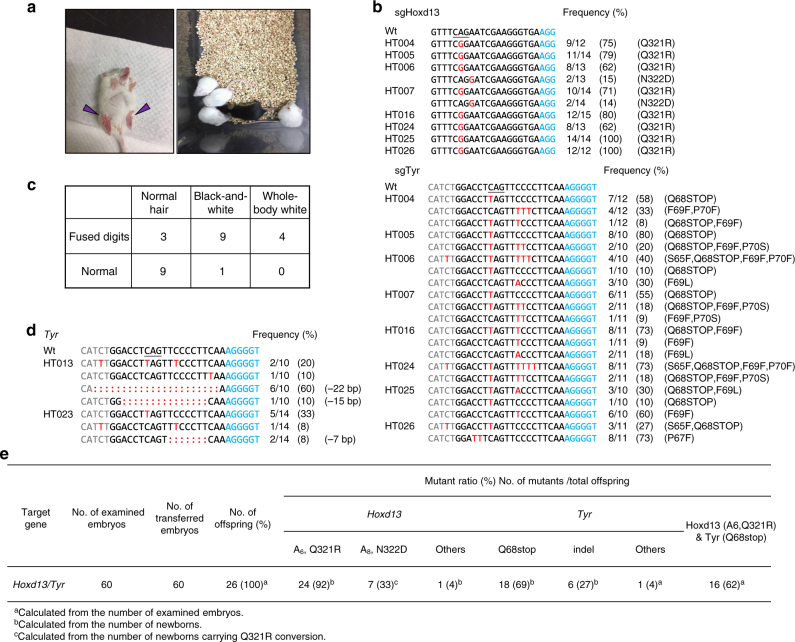
The generation of *Hoxd13* and *Tyr* mutant mouse models by SaBE3 and SpABE. **a** Fused digits and white hair phenotypes in founder mice. Left: Mutant mouse shows whole-body white hair and fused digits (purple arrowhead). Right: all the four mice displayed whole-body white hair and fused digits. **b** Representative alignments of modified sequences from newborn pups after microinjection of SpABE/SaBE3 mRNA and sgRNAs targeting *Hoxd13* and *Tyr* simultaneously. The PAM sequences and substitutions are highlighted in blue and red, respectively; N/N indicates positive colonies out of the total sequenced. The corresponding targeted codons are shown. **c** Summary of phenotypes. Three mice displayed normal hair and fused digits; nine mice displayed black-and-white hair and fused digits; four mice displayed whole-body white hair and fused digits; nine mice displayed normal hair and normal digits; one mouse displayed black-and-white hair and normal digits; no mice displayed whole-body white hair and normal digits. **d** Representative alignments of modified sequences from newborn pups harboring indel. The PAM sequences and substitutions are highlighted in blue and red, respectively; N/N indicates positive colonies out of the total sequenced. The corresponding targeted codons are shown. **e** Summary of the genotyping of *Hoxd13* and *Tyr* mutations of newborns by SaBE3 and SpABE-mediated base editing

These results prompted us to generate mice harboring different modifications at *Hoxd13* and *Tyr*. We injected the zygotes as above and transferred 60 injected embryos into 3 surrogates, obtaining 26 pups (Fig. 3e). At *Hoxd13*, 24 of the pups harbored expected conversion at position 6 (Q321R), of which 7 mice (Founder HT006, HT007, HT012–014, HT017, and HT020) harbored additional A-to-G substitution at position 8, with Founder H020 carrying an additional substitution at position 9 (Fig. 3b, e). Besides, HT021 only harbored A-to-G substitution at position 9. At *Tyr*, 18 out of the 26 (69%) mice harbored Q68stop, among which 16 simultaneously harbored Q321R in *Hoxd13* (Fig. 3b, e). Besides, 7 out of the 26 (27%) mice harbored indels (at the frequencies from 8 to 70%) at *Tyr* (Fig. 3d, e).

Consistent with the genotypes, nine mice had fused digits and black-and-white hair (indicating mosaicism), and four mice had fused digits with uniform white hair (Fig. 3a, c). These data demonstrated the feasibility of modeling complex diseases resulting from combined effects of multiple types of base substitutions.

### Orthogonality analysis of SpABE and SaBE3

We further characterized the multiple types of base editing by assessing the orthogonality of different BEs. To this end, five mice for SpABE alone (H008, H012, H020, H025, and H029) and five mice for SaBE/SpABE double editors (HT006, HT007, HT009, HT011, and HT023) were randomly chosen for deep sequencing for on-/off-target sites. A total of ~100,000–2,900,000 reads for each site were generated, and frequencies of base substitution and indels were calculated (Supplementary Fig. 6a, b). No significant difference in spectrum of mutations, including base substitution and indels, was observed, indicating that ABE is not affected by BE.

### Off-target analysis by deep sequencing and WGS

**Fig. 4:**
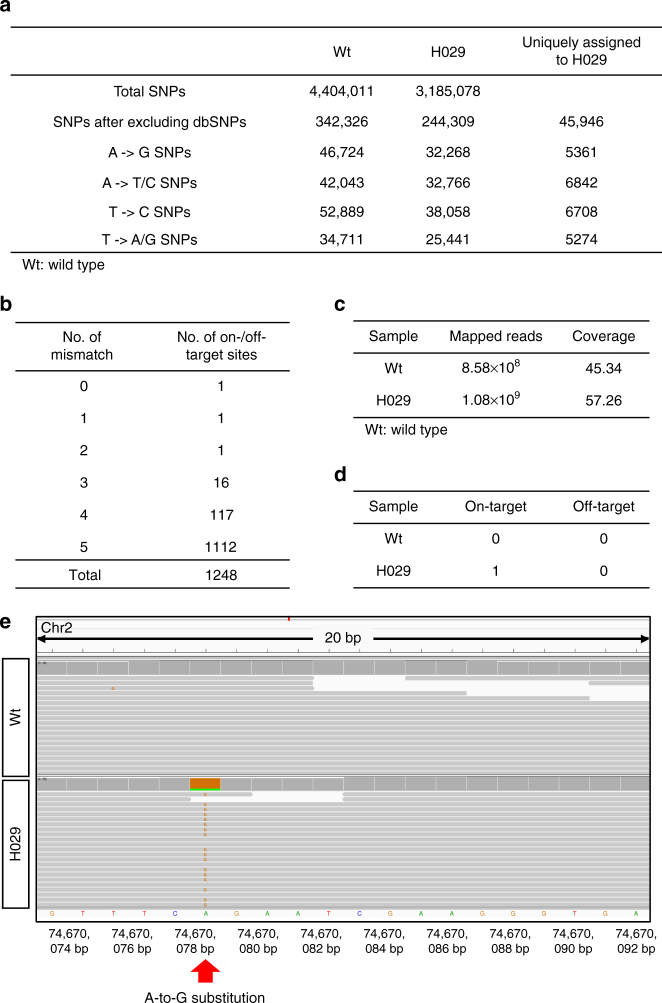
Whole-genome analysis of *Hoxd13* (H029) mutant and wild-type (Wt) mice. **a** Summary of genome sequencing analysis. A *Hoxd13* mutant mouse (H029) and a wild-type mouse (Wt) were sequenced separately using Illumina HiSeq X Ten. A total of 4,404,011 and 3,185,078 SNPs were identified for H029 and Wt, respectively. After filtering out dbSNP (naturally occurring variants in the SNP database), 45,946 SNPs were obtained over the H029 genome. The unwanted base substitutions (A→T/C SNPs, T→A/G SNPs) were excluded next. Then the sequences at the remaining SNP sites were compared with all on-/off-target sequences (20 bp). **b** Summary of on-/off-target site information. A total of 1248 sites, including 1 on-target site and 1, 1, 16, 117, and 1112 off-target sites with 1, 2, 3, 4, and 5 mismatch/es, respectively, were analyzed. **c** Summary of the whole-genome sequencing. **d** Summary of off-target analysis. After comparing the sequences at the remaining SNP sites with the 1248 on-/off-target sequence (20 bp), the A-to-G substitution was only detected within the on-target sequencing in H029. **e** Confirmation of the on-target mutation by the analysis of whole-genome sequencing. Red arrow indicates the A-to-G substitution within 20 bp on-target sequence in H029

Finally, we further evaluated the off-target effects of SpABE and SaBE3 by amplicon-based deep sequencing. We first utilized the online tool (http://www.rgenome.net/cas-offinder/) to find 20, 19, and 18 potential off-target sites in the genome with up to 3-nucleotide mismatches for sgAr-1/sgAr-15/sgHoxd13 and 42 (up to 4-nucleotide mismatches) for sgTyr^20^. We randomly chose 11, 12, 10, and 11 potential off-target sites for sgAr-1, sgAr-15, sgHoxd13, and sgTyr, respectively (Supplementary Fig. 7a–d). These sites were sequenced using tail DNA from three mice per sgRNA (A001, A004, and A015 for sgAr-1; A047, A052, and A063 for sgAr-15; H008, H012, and H029 for sgHoxd13; HT006, HT007, and HT009 for sgTyr). No base substitution was detected at any of these sites in a total of ~14,800,000 reads. Our data confirmed the precision of base editing in vivo by both BEs. To further explore the precision of ABE-mediated base editing, a WGS was performed using genomic DNA from a *Hoxd13* mutant mouse (H029) and a wild-type mouse (Wt) as the control at depth of about 45–57× (Fig. 4c). A total of 4,404,011 and 3,185,078 single-nucleotide polymorphisms (SNPs) were detected for Wt and H029, respectively. After filtering out dbSNP (naturally occurring variants in the SNP database), 45,946 SNPs were obtained over the H029 genome. We next excluded the unwanted base substitutions (A→T/C SNPs, T→A/G SNPs) as shown in Fig. 4a and then compared the sequences around the remaining SNP sites with all on-/off-target sequences (20 bp). We analyzed a total of 1248 sites, including 1 on-target site and 1, 1, 16, 117, and 1112 off-target sites with 1, 2, 3, 4, and 5 mismatch/es, respectively (Fig. 4b). Only the A-to-G substitution within the target window was observed (Fig. 4d, e). Taken together, both deep sequencing and WGS demonstrated the precision of ABE-mediated base editing in vivo.

### Successful germline transmission of base editing

To determine whether the mutations are heritable, we mated the male founder with fused digits (H017) to a Wt female, producing 8 pups (5 males and 3 females). All pups harbored mutation at position A_5_ with the frequency of about 50% (Supplementary Fig. 8a), demonstrating germline transmission of the mutation introduced by ABE.

## Discussion

ABE converts A-to-G, which has obvious clinical implications^6^. To demonstrate its utility in vivo, we microinjected ABE mRNA together with sgRNAs into zygotes and achieved successful A-to-G conversion with an efficiency of up to 100% at *Ar* and *Hoxd13* loci in embryos and mice. The mutations are of clinical relevance, and the mutant mice recapitulated the phenotypes of AIS (sex reversal) and Syndactyly (fused digits) from the *Ar* and *Hoxd13* mutations, respectively. These results confirmed the power of ABE in base editing in vivo.

Considering that different kinds of mutations, including A-to-G and C-to-T, usually co-exist in vast majority of genetic diseases, we sought to simultaneously create A-to-G and C-to-T substitutions using SpABE together with SaBE3, which recognize different PAM sequences. Compound mutations were indeed generated, and furthermore, the two editors are fully orthogonal to each other, indicating that they can be safely used in combination.

To further analyze the precision of ABE-mediated base editing, we performed comprehensive on- and off-target analysis by PCR-based deep sequencing and WGS. No off-target effect was detected, and A-to-G substitution occurred exclusively within the target window, indicating the precision of ABE-mediated base editing in vivo.

In summary, using SpABE alone or in combination with SaBE3, we demonstrated the feasibility of introducing simple and compound clinical modifications and recapitulating relevant defects. Furthermore, the efficiency and precision of base editing in vivo opens the potential for correcting the human genetic disease using BEs.

## Methods

### Animals

Mice were housed in standard cages in an Assessment and Accreditation of Laboratory Animal Care credited specific pathogen-free animal facility under a 12:12 h light/dark cycle. All the experiment procedures were approved by the Animal Care and Use Committee of the Institute of Neuroscience, Chinese Academy of Sciences, Shanghai, China.

### Plasmids construction

For construction of sgRNAs, oligos were synthesized, annealed, and cloned into BsaI site of the sgRNA expression vector. Plasmids used include pGL3-U6-sgRNA-PGK-puromycin (Addgene, 51133), pUC57-sgRNA expression vector (Addgene, 51132), pCMV-SaBE3 (Addgene, 85169), pGFP-N1 (Addgene, 54712), pUC57-Sa sgRNA expression vector, pGL3-U6-sgRNA-EGFP, pGL3-U6-sgRNA-BFP, and pCMV-ABE.

### Cell culture and transfection

N2a cells were purchased from ATCC (ATCC, CCL-131) and cultured in Dulbecco’s Modified Eagle Medium (Hyclone), supplemented with 10% fetal bovine serum (v/v) (Gemini) and 1% Penicillin Streptomycin (v/v) (Gibco). Cells were transfected using Lipofectamine 2000 reagent (Life Technologies) according to the manufacturer’s protocols. In brief, N2a cells were seeded on poly-D-lysine-coated 12-well plates (JETBIOFIL), transfection were performed at approximately 70% density about 12 h after seeding, and 1000 ng sgRNA and 1000 ng ABE plasmids were transfected with 4 μl Lipofectamine 2000. GFP-positive cells were harvested from FACS 72 h after transfection. Cells were incubated at 37 °C with 5% CO_2_. sgRNAs used are listed in Supplementary Table 1.

### Genomic DNA extraction and genotyping

Genomic DNA of cells collected from FACS was extracted using QuickExtract™ DNA Extraction Solution (Lucigen) according to the manufacturer’s protocols, genomic DNA of mouse tail was extracted by phenol–chloroform method, and genomic DNA of zygotes was amplified according to methods described in Methods subsection “Whole-genome random amplification”. Primers used for genotyping are listed in Supplementary Table 2.

### Whole-genome random amplification

Single embryo was transferred to 200 μl tube containing 5 μl of an alkaline lysis solution (200 mM KOH/50 mM dithiothreitol). After a 10-min incubation at 65 °C, 5 μl of neutralization solution (900 mM Tris-HCl, pH 8.3/300 mM KCl/200 mM HCl) was added. The lysed and neutralized sample was added with 5 μl of a 400 μM solution of random primers (Genscript, Nanjing, China), 6 μl of 10× PCR buffer (Takara, Dalian, China), 3 μl of a mixture of the 4 dNTPs (each at 2.5 mM), and 1 μl of Taq polymerase (Takara, Dalian, China) and brought to 60 μl with water. Fifty primer-extension cycles were carried out in a MyCycler thermo-cycler (Bio-Rad, USA). Each cycle consisted of a 1-min denaturation step at 92 °C, a 2-min annealing step at 37 °C, a programmed ramping step of 10 s/degree to 55 °C, and a 4-min incubation at 55 °C for polymerase extension. Then the products were used as the PCR templates.

### Flow cytometry

Cells were harvested and subjected to flow cytometry 72 h after transfection. sgRNAs were annealed into pGL3-U6-sgRNA-PGK-puromycin, and BFP signal was detected with flow cytometry. ABE, sgRNA, and pGFP-N1 plasmids were transfected simultaneously. Scramble sgRNA and sgRNA-BFP were transfected as control. A total of 10,000 cell events were collected and analyzed in the FlowJo software.

### In vitro transcription

In brief, pCMV-ABE/pCMV-SaBE3 vector were linearized by BbsI enzyme (NEB) and in vitro transcribed using the T7 Ultra Kit (Ambion) according to the manufacturer’s protocols. SpABE/SaBE3 mRNA was purified by the Mini Kit (Qiagen). sgRNA oligos were annealed into pUC57-sgRNA expression vectors with T7 promoter. Then sgRNAs were amplified and transcribed in vitro by the MEGAshortscript Kit (Ambion). The sgRNAs were purified by the MEGAclear Kit (Ambion) according to the manufacturer’s protocols. Primers used for transcription in vitro are listed in Supplementary Table 3.

### Microinjection of one-cell embryos and embryo transfer

Female C57BL/6 mice (4-week-old) were superovulated and mated to C57BL/6 male mice. Zygotes were collected from oviducts of the female mice. mRNA mixtures containing sgRNA and SpABE/SaBE3 were injected into the cytoplasm of zygotes in a droplet of M2 medium containing 5 µg/ml cytochalasin B using a piezo (Primetech) microinjector. The injected zygotes were cultured in KSOM mediums at 37 °C under 5% CO_2_ in air and transferred to oviducts of pseudopregnant ICR females at 0.5 days post copulation. The concentration of mRNA used for injection were 100 and 50 ng/µl for BE3/SaBE3 and sgRNA, respectively.

### Targeted deep sequencing

Potential off-target sites were predicted by Cas-OFFinder (http://www.rgenome.net/cas-offinder)^20^. The on-target and off-target sites were amplified from mouse genomic DNA using Phanta® Max Super-Fidelity DNA Polymerase (Vazyme). The paired-end sequencing of PCR amplicons was performed by Illumina Nextseq 500 (2 × 150) platform at CAS-MPG Partner Institute for Computational Biology Omics Core, Shanghai, China. BWA and Samtools were employed for mapping the pair-end reads to mouse genome mm10, and VarDict was used to call single-nucleotide variants and insertions and deletions (indels) in amplicon aware mode. The aligned reads were visualized by using the Integrated Genome Viewer and tabbed using Pysamstats^21–24^. Primers used for targeted deep sequencing are listed in Supplementary Table 3.

### Whole-genome sequencing

Genomic DNA was extracted by phenol–chloroform method. In all, 100 ng DNA was fragmented using a Covaris LE220 (Covaris), size selected (300–550 bp), end-repaired, A-tailed, and adapter ligated. Libraries were sequenced using the Hiseq X Ten platform (Illumina) as paired-end 150 base reads at the CAS-MPG Partner Institute for Computational Biology Omics Core, Shanghai, China.

### Statistical analysis

No statistical methods were used to predetermine sample size for in vitro or in vivo experiments. Data are shown as mean ± s.e.m. unless stated otherwise. Statistical analysis is performed by two-tailed Student’s *t*-test. Statistical analysis was performed in Graph Pad PRISM 7.

### Data availability

High-throughput sequencing data have been deposited in the NCBI Sequence Read Archive database under accession code (SRP140663). Plasmids pUC57-Sa sgRNA (107720) expression vector, pGL3-U6-sgRNA-EGFP (107721), pGL3-U6-sgRNA-BFP (107722), and pCMV-ABE (107723) are available from Addgene. All other data are available upon reasonable request.

## Electronic supplementary material

Supplementary Information
