# Supplementary material for: Efficient generation of mouse models of human diseases via ABE- and BE-mediated base editing
Source: Nat Commun. 2018 Jun 14;9:2338. doi: 10.1038/s41467-018-04768-7 (PMC6002399; doi:10.1038/s41467-018-04768-7)
Supplement: Supplementary file 1 — Supplementary Information [file 41467_2018_4768_MOESM1_ESM.pdf]

Efficient generation of mouse models of human diseases via ABE- and BE-mediated base editing

SUPPLEMENTARY INFORMATION

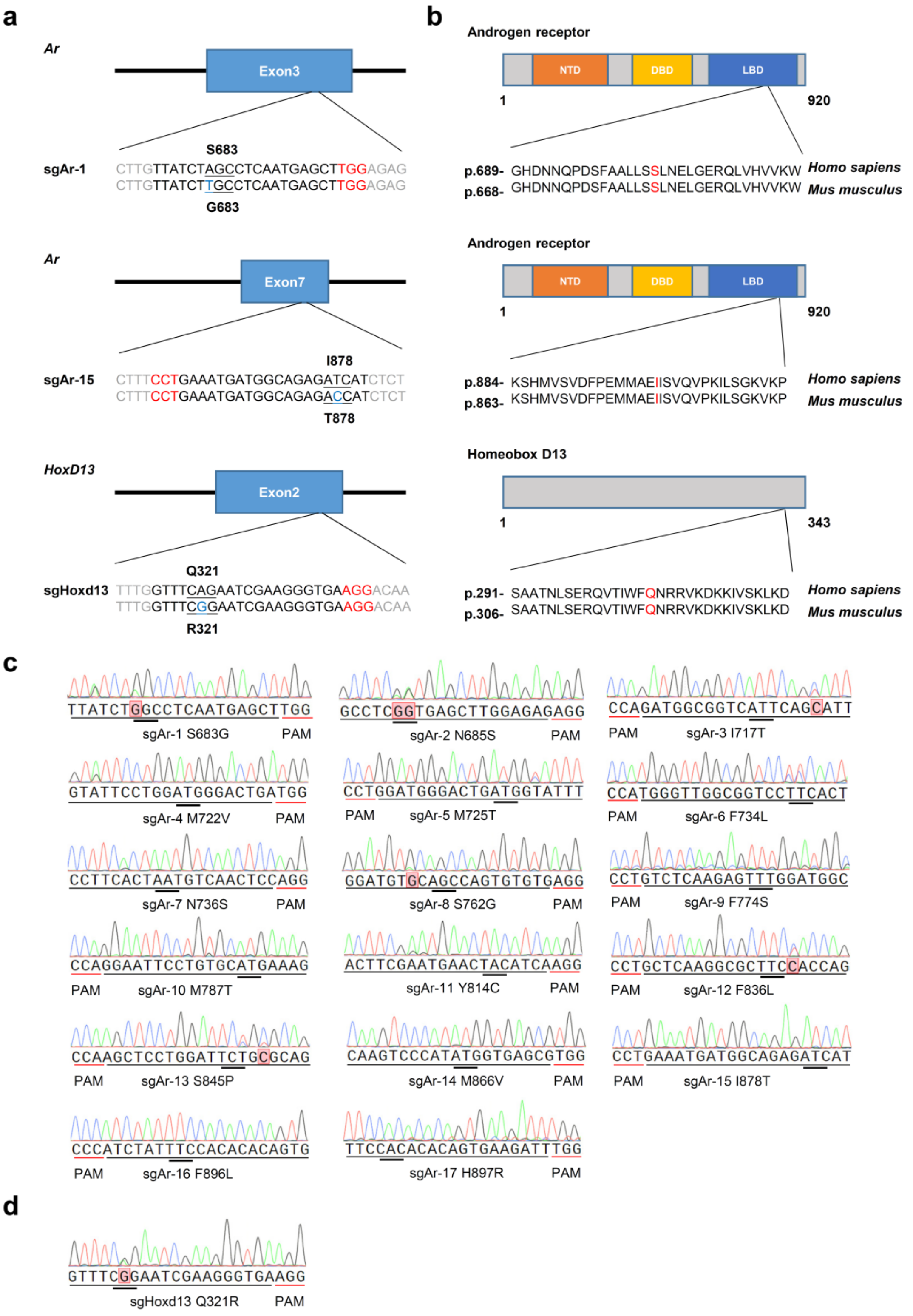

Supplementary Figure 1. The targeted sequences at the *Ar* and *Hoxd13* loci.

(a) The PAM sequences and the sgRNA targeted sequences are shown in red and black and underlined, respectively; the substitutions in blue.

(b) Schematic illustration shows the localization of disease relevant mutation sits on Androgen receptor protein and Homeobox D13 protein of human. Lower parts of each illustration show the alignments of the conserved amino acid sequence from *H. sapiens* to *M. musculus*. The targeted

sites in this study were highlighted in red.

(c) Sanger sequencing chromatograms of ABE-mediated base editing of *Ar* in N2a cells. The PAM sequence and sgRNA sequence are underlined in red and black, respectively. The corresponding targeted codons are shown under the target sequence.

(d) Sanger sequencing chromatograms of ABE-mediated base editing of *Hoxd13* in N2a cells. The PAM sequence and sgRNA sequence are underlined in red and black, respectively. The corresponding targeted codons are shown under the target sequence.

a

sgAr-1		Frequency(%)	
WT	TTATCTAGCCTCAATGAGCTTGG		
#1	TTGTCTGGCCTCAATGAGCTTGG	8/15	(53)
	TTGTCTAGCCTCAATGAGCTTGG	4/15	(27)
#2	TTGTCTGGCCTCAATGAGCTTGG	2/11	(18)
	TTGTCTAGCCTCAATGAGCTTGG	1/11	(9)
#3	TTGTCTGGCCTCAATGAGCTTGG	4/13	(31)
	TTGTCTAGCCTCAATGAGCTTGG	5/13	(38)
#4	TTGTCTGGCCTCAATGAGCTTGG	12/13	(92)
	TTATCTGGCCTCAATGAGCTTGG	1/13	(8)
#5	TTGTCTGGCCTCAATGAGCTTGG	7/11	(64)
	TTATCTGGCCTCAATGAGCTTGG	4/11	(36)
#6	TTATCTGGCCTCAATGAGCTTGG	4/12	(33)
	TTGTCTGGCCTCAATGAGCTTGG	5/12	(42)
	TTGTCTAGCCTCAATGAGCTTGG	1/12	(8)
#7	TTGTGTGGCCTCAATGAGCTTGG	2/15	(13)
	TTGTCTGGCCTCAATGAGCTTGG	12/15	(80)
	TTATCTGGCCTCAATGAGCTTGG	1/15	(7)
#8	TTGTCTGGCCTCAATGAGCTTGG	4/10	(40)
	TTGTCTAGCCTCAATGAGCTTGG	3/10	(30)
#9	TTGTCTGGCCTCAATGAGCTTGG	11/11	(100)
#10	TTATCTGGCCTCAATGAGCTTGG	4/17	(24)
	TTGTCTGGCCTCAATGAGCTTGG	10/17	(59)
	TTGTCTAGCCTCAATGAGCTTGG	2/17	(12)
#11	TTATCTGGCCTCAATGAGCTTGG	11/11	(100)
#12	TTGTCTGGCCTCAATGAGCTTGG	2/12	(17)
	TTATCTGGCCTCAATGAGCTTGG	3/12	(25)
	TTGTCTAGCCTCAATGAGCTTGG	4/12	(33)
	TTATATGGCCTCAATGAGCTTGG	1/12	(8)
#13	TTGTCTGGCCTCAATGAGCTTGG	9/16	(56)
	TTATCTGGCCTCAATGAGCTTGG	3/16	(19)
	TTGTCTAGCCTCAATGAGCTTGG	3/16	(19)
#14	TTGTCTGGCCTCAATGAGCTTGG	10/11	(91)
#15	TTGTCTGGCCTCAATGAGCTTGG	10/13	(77)
	TTATCTGGCCTCAATGAGCTTGG	1/13	(8)
	TTGTCTAGCCTCAATGAGCTTGG	1/13	(8)
#16	TTGTCTGGCCTCAATGAGCTTGG	6/10	(60)
	TTATCTGGCCTCAATGAGCTTGG	2/10	(20)
	TTATCTGGCCCCAATGAGCTTGG	1/10	(10)

b

sgAr-15		Frequency(%)	
WT	CCTGAAATGATGGCAGAGATCAT		
#1	CCTGAAATGATGGCAGAGACCAT	5/13	(38)
#2	CCTGAAATGATGGCAGAGACCAT	1/12	(8)
#3	CCTGAAATGATGGCAGAGACCAT	7/15	(47)
#4	CCTGAAATGATGGCAGAGACCAT	7/14	(50)
#5	CCTGAAATGATGGCAGAGACCAT	4/10	(40)
#6	CCTGAAATGATGGCAGAGACCAT	2/10	(20)
#7	CCTGAAATGATGGCAGAGACCAT	6/14	(43)
#8	CCTGAAATGATGGCAGAGACCAT	7/13	(54)
#9	CCTGAAATGATGGCAGAGACCAT	4/10	(40)
#10	CCTGAAATGATGGCAGAGACCAT	5/13	(38)
#11	CCTGAAATGATGGCAGAGACCAT	6/12	(50)
#12	CCTGAAATGATGGCAGAGACCAT	7/14	(50)
#13	CCTGAAATGATGGCAGAGACCAT	10/14	(71)
#14	CCTGAAATGATGGCAGAGACCAT	5/12	(42)
#15	CCTGAAATGATGGCAGAGACCAT	3/12	(25)
#16	CCTGAAATGATGGCAGAGACCAT	6/11	(55)

c

sgHoxd13		Frequency(%)	
WT	GTTTCAGAATCGAAGGGTGAAGG		
#1	GTTTCGGAATCGAAGGGTGAAGG	8/13	(62)
	GTTTCAGGATCGAAGGGTGAAGG	4/13	(31)
#2	GTTTCGGAATCGAAGGGTGAAGG	7/10	(70)
	GTTTCGGGATCGAAGGGTGAAGG	3/10	(30)
#3	GTTTCGGAATCGAAGGGTGAAGG	10/15	(67)
#4	GTTTCGGAATCGAAGGGTGAAGG	4/12	(33)
#5	GTTTCGGAATCGAAGGGTGAAGG	2/11	(18)
	GTTTCAGGATCGAAGGGTGAAGG	5/11	(45)
#6	GTTTCGGAATCGAAGGGTGAAGG	4/12	(33)
	GTTTCAGGATCGAAGGGTGAAGG	1/12	(8)
#7	GTTTCGGAATCGAAGGGTGAAGG	9/10	(90)
#8	GTTTCGGAATCGAAGGGTGAAGG	9/12	(75)
#9	GTTTCGGAATCGAAGGGTGAAGG	5/12	(42)
	GTTTCGGAGTCGAAGGGTGAAGG	2/12	(17)
	GTTTCAGGATCGAAGGGTGAAGG	2/12	(17)
#10	GTTTCGGAATCGAAGGGTGAAGG	6/10	(60)
#11	GTTTCGGAATCGAAGGGTGAAGG	9/10	(90)
#12	GTTTCGGAATCGAAGGGTGAAGG	9/10	(90)
#13	GTTTCGGAATCGAAGGGTGAAGG	10/10	(100)
#14	GTTTCGGAATCGAAGGGTGAAGG	6/11	(55)
	GTTTCAGGATCGAAGGGTGAAGG	5/11	(45)

d

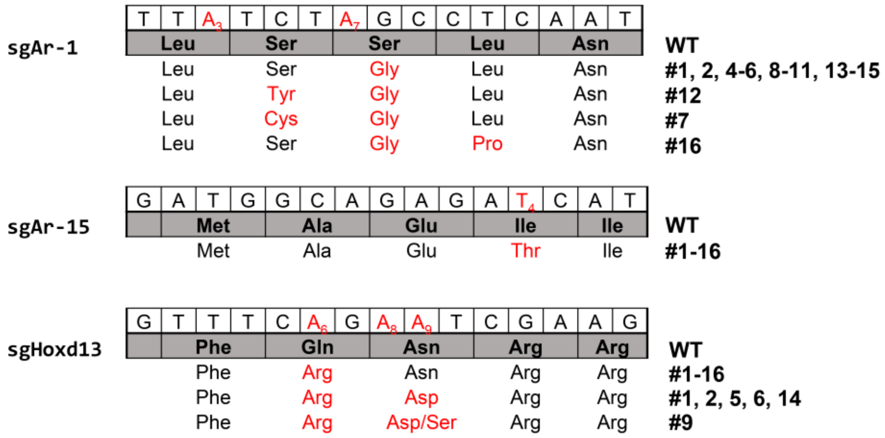

e

Target gene	Methods		No. of examined embryos	No. of blastocysts (%)	Mutant ratio (%)	
					No. of mutants /tested blastocysts	No. of indels/ total mutants
Ar	sgAr-1+ABE mRNA	Microinjection	18	17 (94)[a]	16/16 (100)	0/16(0)
Ar	sgAr-15+ABE mRNA	Microinjection	22	20 (91)[a]	16/16 (100)	0/16(0)
Hoxd13	sgHoxd13+ABE mRNA	Microinjection	14	13 (93)[a]	14/14 (100)	0/14(0)

[a]Calculated from the number of examined embryos.

Supplementary Figure 2. The sequencing analysis of the embryos by ABE-mediated base editing.

(a-c) Alignments of modified sequences from embryos after microinjection of ABE mRNA and sgRNAs into one-cell embryos. The PAM sequence and substitutions are highlighted in blue and red, respectively; N/N indicates positive colonies out of total sequenced. The corresponding targeted codons are shown. (a) The sequencing results for sgAr-1; (b) The sequencing results for sgAr-15; (c) The sequencing results for sgHoxd13.

(d) Alignments of modified amino acid sequences and their corresponding number of the samples.

(e) Summary of the manipulation and genotyping of mouse embryos.

a

sgAr-1		Frequency(%)	
WT	TTATCTAGCCTCAATGAGCTTGG		
A001	TTGTCTGGCCTCAATGAGCTTGG	10/11	(91)
	TTGTCTAGCCTCAATGAGCTTGG	1/11	(9)
A002	TTGTCTGGCCTCAATGAGCTTGG	2/11	(18)
A003	TTGTCTAGCCTCAATGAGCTTGG	10/10	(100)
A004	TTGTCTGGCCTCAATGAGCTTGG	10/11	(91)
	TTATCTGGCCTCAATGAGCTTGG	1/11	(9)
A005	TTATCTGGCCTCAATGAGCTTGG	3/12	(25)
	TTGTCTAGCCTCAATGAGCTTGG	3/12	(25)
A006	TTATCTGGCCTCAATGAGCTTGG	5/13	(38)
	TTGTCTGGCCTCAATGAGCTTGG	4/13	(31)
	TTGTCTAGCCTCAATGAGCTTGG	3/13	(23)
A007	TTATCTGGCCTCAATGAGCTTGG	6/11	(55)
	TTGTCTGGCCTCAATGAGCTTGG	1/11	(9)
A008	TTATCTGGCCTCAATGAGCTTGG	8/12	(67)
	TTGTCTGGCCTCAATGAGCTTGG	1/12	(8)
	TTGTCTAGCCTCAATGAGCTTGG	3/12	(25)
A009	TTGTCTAGCCTCAATGAGCTTGG	11/12	(92)
	TTGTCTGGCCTCAATGAGCTTGG	1/12	(8)
A010	TTGTCTGGCCTCAATGAGCTTGG	5/12	(42)
A011	TTGTCTGGCCTCAATGAGCTTGG	1/10	(90)
	TTGTCTAGCCTCAATGAGCTTGG	9/10	(10)
A012	TTGTCTGGCCTCAATGAGCTTGG	11/15	(73)
A013	TTGTCTGGCCTCAATGAGCTTGG	11/12	(92)
A014	TTGTCTGGCCTCAATGAGCTTGG	13/13	(100)
A015	TTGTCTGGCCTCAATGAGCTTGG	11/11	(100)
A016	TTGTCTGGCCTCAATGAGCTTGG	11/12	(92)
	TTGTCTAGCCTCAATGAGCTTGG	1/12	(8)
A017	TTGTCTGGCCTCAATGAGCTTGG	5/10	(50)
	TTATCTGGCCTCAATGAGCTTGG	2/10	(20)
A018	TTATCTGGCCTCAATGAGCTTGG	3/10	(30)
	TTGTCTAGCCTCAATGAGCTTGG	5/10	(50)
A019	TTGTCTGGCCTCAATGAGCTTGG	3/11	(27)
	TTATCTGGCCTCAATGAGCTTGG	7/11	(64)
A020	TTGTCTGGCCTCAATGAGCTTGG	3/13	(23)
	TTGTTTGGCCTCAATGAGCTTGG	1/13	(8)
A021	TTGTCTGGCCTCAATGAGCTTGG	11/11	(100)
A022	TTGTCTGGCCTCAATGAGCTTGG	8/13	(62)
	TTATCTGGCCTCAATGAGCTTGG	4/13	(30)
	TTGTCTAGCCTCAATGAGCTTGG	1/13	(8)

b

sgAr-15		Frequency(%)	
WT	CCTGAAATGATGGCAGAGATCAT		
A046	CCTGAAATGATGGCAGAGACCAT	9/12	(75)
A047	CCTGAAATGATGGCAGAGACCAT	11/12	(92)
A048	CCTGAAATGATGGCAGAGACCAT	6/10	(60)
A049	CCTGAAATGATGGCAGAGATCAT	11/11	(100)
A050	CCTGAAATGATGGCAGAGACCAT	6/12	(50)
A051	CCTGAAATGATGGCAGAGACCAT	4/10	(40)
A052	CCTGAAATGATGGCAGAGACCAT	10/10	(100)
A053	CCTGAAATGATGGCAGAGACCAT	5/12	(42)
A054	CCTGAAATGATGGCAGAGACCAT	12/12	(100)
A055	CCTGAAATGATGGCAGAGACCAT	5/10	(50)
A056	CCTGAAATGATGGCAGAGACCAT	3/11	(27)
A057	CCTGAAATGATGGCAGAGACCAT	5/11	(45)
A058	CCTGAAATGATGGCAGAGACCAT	3/12	(25)
A059	CCTGAAATGATGGCAGAGACCAT	3/11	(27)
A060	CCTGAAATGATGGCAGAGACCAT	4/11	(36)
A061	CCTGAAATGATGGCAGAGACCAT	4/13	(31)
A062	CCTGAAATGATGGCAGAGACCAT	6/11	(55)
A063	CCTGAAATGATGGCAGAGACCAT	12/12	(100)
A064	CCTGAAATGATGGCAGAGACCAT	3/12	(25)

c

sgHoxd13		Frequency(%)	
WT	GTTTCAGAATCGAAGGGTGAAGG		
H001	GTTTCGGAATCGAAGGGTGAAGG	8/20	(40)
	GTTTCAGGGTCGAAGGGTGAAGG	5/20	(25)
H002	GTTTCAGAATCGAAGGGTGAAGG	13/13	(100)
H003	GTTTCGGAATCGAAGGGTGAAGG	2/13	(15)
	GTTTCAGGATCGAAGGGTGAAGG	8/13	(62)
H004	GTTTCAGAATCGAAGGGTGAAGG	10/10	(100)
H005	GTTTCAGAATCGAAGGGTGAAGG	12/12	(100)
H006	GTTTCGGAATCGAAGGGTGAAGG	3/11	(27)
H007	GTTTCGGAATCGAAGGGTGAAGG	4/10	(40)
H008	GTTTCGGAATCGAAGGGTGAAGG	6/10	(60)
H009	GTTTCGGAATCGAAGGGTGAAGG	6/15	(40)
H010	GTTTCGGAATCGAAGGGTGAAGG	3/11	(27)
H011	GTTTCGGAATCGAAGGGTGAAGG	2/20	(10)
	GTTTCAGGATCGAAGGGTGAAGG	8/20	(40)
H012	GTTTCGGAATCGAAGGGTGAAGG	9/10	(90)
H013	GTTTCGGAATCGAAGGGTGAAGG	1/12	(8)
	GTTTCGGGATCGAAGGGTGAAGG	2/12	(17)
H014	GTTTCGGAATCGAAGGGTGAAGG	5/11	(45)
H015	GTTTCGGAATCGAAGGGTGAAGG	8/10	(80)
	GTTTCAGGATCGAAGGGTGAAGG	1/10	(10)

sgHoxd13		Frequency(%)	
WT	GTTTCAGAATCGAAGGGTGAAGG		
H016	GTTTCAGAATCGAAGGGTGAAGG	10/10	(100)
H017	GTTTCGGAATCGAAGGGTGAAGG	8/11	(73)
H018	GTTTCGGAATCGAAGGGTGAAGG	9/13	(69)
	GTTTCGGGATCGAAGGGTGAAGG	4/13	(31)
H019	GTTTCAGAATCGAAGGGTGAAGG	13/13	(100)
H020	GTTTCGGAATCGAAGGGTGAAGG	9/10	(90)
H021	GTTTCGGAATCGAAGGGTGAAGG	9/12	(75)
H022	GTTTCGGAATCGAAGGGTGAAGG	12/12	(100)
H023	GTTTCGGAATCGAAGGGTGAAGG	4/10	(40)
	GTTTCGGGATCGAAGGGTGAAGG	5/10	(50)
H024	GTTTCGGAATCGAAGGGTGAAGG	4/10	(40)
	GTTTCAGGATCGAAGGGTGAAGG	5/10	(50)
H025	GTTTCGGAATCGAAGGGTGAAGG	8/10	(80)
H026	GTTTCGGAATCGAAGGGTGAAGG	7/13	(54)
H027	GTTTCGGAATCGAAGGGTGAAGG	2/11	(18)
H028	GTTTCAGAATCGAAGGGTGAAGG	11/11	(100)
H029	GTTTCGGAATCGAAGGGTGAAGG	12/12	(100)
H030	GTTTCGGAATCGAAGGGTGAAGG	4/11	(36)
	GTTTCAGGATCGAAGGGTGAAGG	2/11	(18)
H031	GTTTCGGAATCGAAGGGTGAAGG	4/18	(22)
	GTTTCGGGATCGAAGGGTGAAGG	5/18	(27)
H032	GTTTCGGAATCGAAGGGTGAAGG	6/12	(50)
	GTTTCGGGATCGAAGGGTGAAGG	6/12	(50)

d

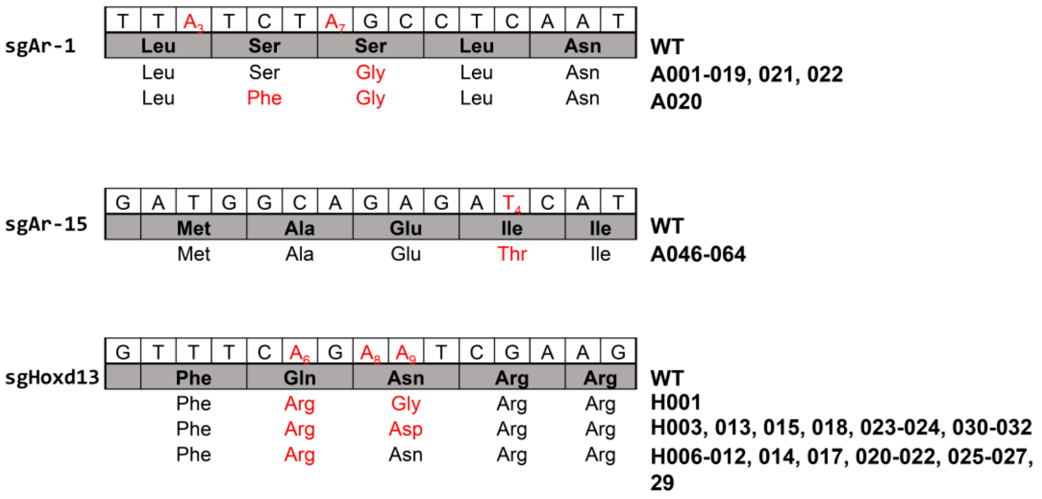

Supplementary Figure 3. The sequencing analysis of the newborn pups targeting at *Ar* and *Hoxd13*, respectively.

(a-c) Alignments of modified sequences from newborn pups after microinjection of ABE mRNA and sgRNAs targeting at *Ar* and *Hoxd13* respectively into one-cell embryos. The PAM sequences and substitutions are highlighted in blue and red, respectively; N/N indicates positive colonies out of total sequenced. The corresponding targeted codons are shown. (a) The sequencing results for sgAr-1; (b) The sequencing results for sgAr-15; (c) The sequencing results for sgHoxd13.

(d) Alignments of modified amino acid sequences and their corresponding number of the tested pups.

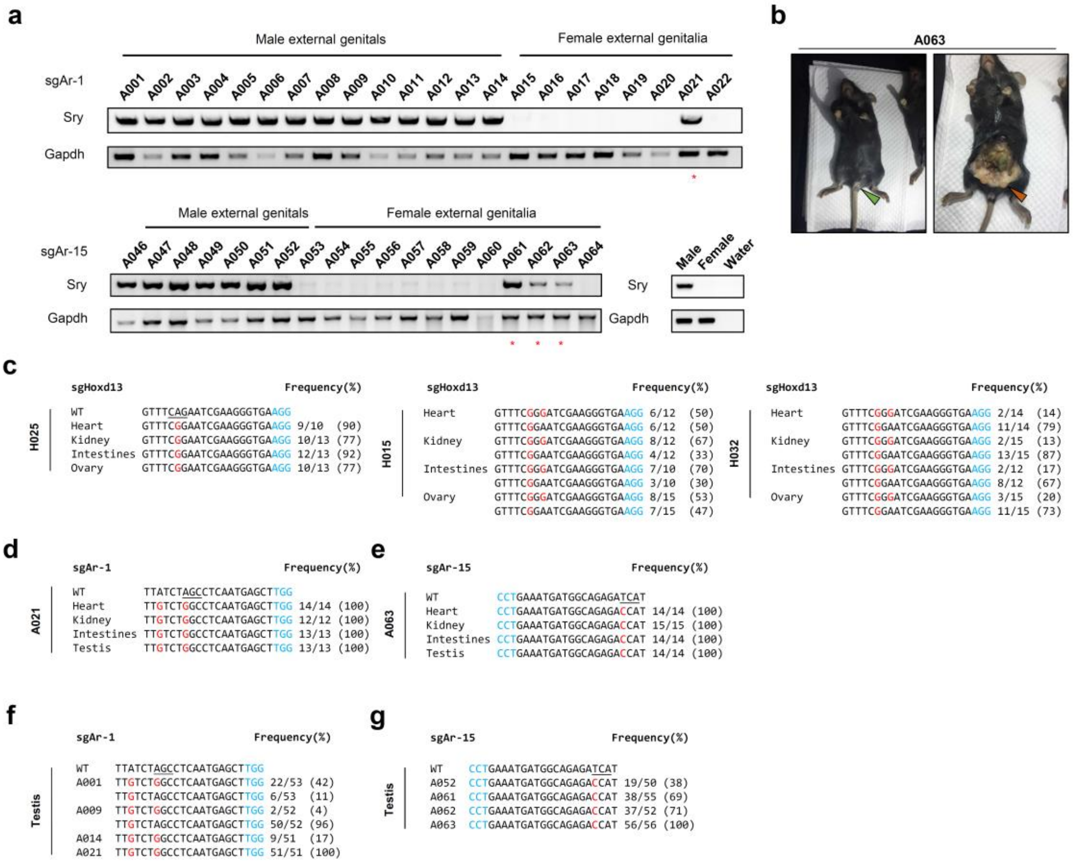

Supplementary Figure 4. Phenotype and genotype analysis of founder mice.

(a) PCR-based genotype analysis of gender. The red stars indicate the feminized mice carrying Y chromosome-specific gene, Sry, suggesting sex reversal.

(b) Sexual reverse phenotypes in founder mouse. Left: five-week-old mouse with female genitalia (green arrowhead); Right: A063 mouse with internal genitalia of male (orange arrowhead).

(c) Alignments of modified sequences from different tissues of three mice targeting Hoxd13. The PAM sequences and substitutions are highlighted in blue and red, respectively; N/N indicates positive colonies out of total sequenced. The corresponding targeted codons are shown.

(d-e) Alignments of modified sequences from different tissues of two mice targeting Ar. The PAM sequences and substitutions are highlighted in blue and red, respectively; N/N indicates positive colonies out of total sequenced. The corresponding targeted codons are shown. (d) the sequencing results for Founder A021; (e) the sequencing results for Founder A063.

(f-g) Alignments of modified sequences from testis of mice harboring 100% base substitution or displaying phenotypes. The PAM sequences and substitutions are highlighted in blue and red, respectively; N/N indicates positive colonies out of total sequenced. The corresponding targeted codons are shown. (f) the sequencing results from A001, A009, A014 and A021 for sgAr-1; (g) the sequencing results from A052, A061, A062 and A063 for sgAr-15.

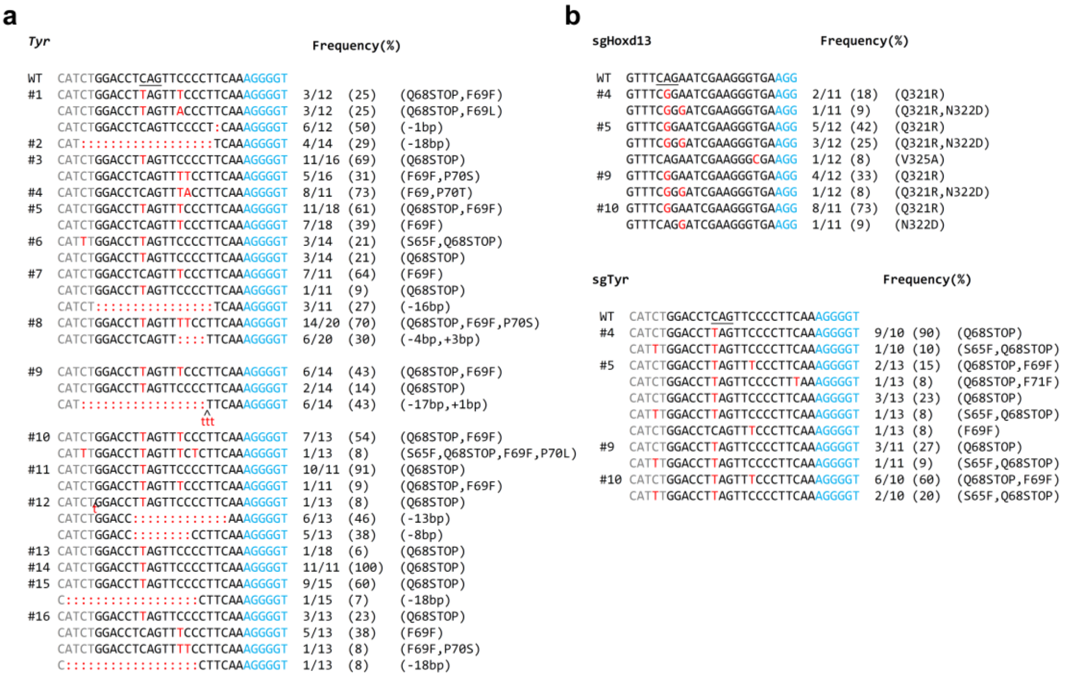

c

Target gene	Methods	No. of examined embryos	No. of blastocysts (%)	Mutant ratio (%) No. of mutants /total embryos						
				Hoxd13			*Tyr*			Hoxd13(A6,Q321R) & Tyr(Q68stop)
				A_6, Q321R	A_8, N322D	Others	Q68stop	indel	Others	
Hoxd13/Tyr	Microinjection	15	15(100) [a]	13/15 (87) [a]	5/15 (33) [a]	0/15 (0) [a]	1/15 (7) [a]	6/15 (40) [a]	7/15 (47) [a]	1/15 (7) [a]

[a]Calculated from the number of examined embryos.

Supplementary Figure 5. The sequencing analysis of the embryos by BE-mediated base editing targeting at *Tyr* and *Tyr/Hoxd13*.

(a) Alignments of modified sequences from embryos after microinjection of SaBE3 mRNA and sgRNAs targeting at *Tyr* into one-cell embryos. The PAM sequences and substitutions are highlighted in blue and red, respectively; N/N indicates positive colonies out of total sequenced. The corresponding targeted codons are shown.

(b) Representative alignments of modified sequences from embryos after microinjection of ABE/saBE3 mRNA and sgRNAs targeting *Hoxd13* and *Tyr*, respectively. The PAM sequences and substitutions are highlighted in blue and red, respectively; N/N indicates positive colonies out of total sequenced. The corresponding targeted codons are shown.

(c) Summary of the manipulation and genotyping of mouse embryos for SaBE3 and ABE targeting *Hoxd13* and *Tyr* simultaneously.

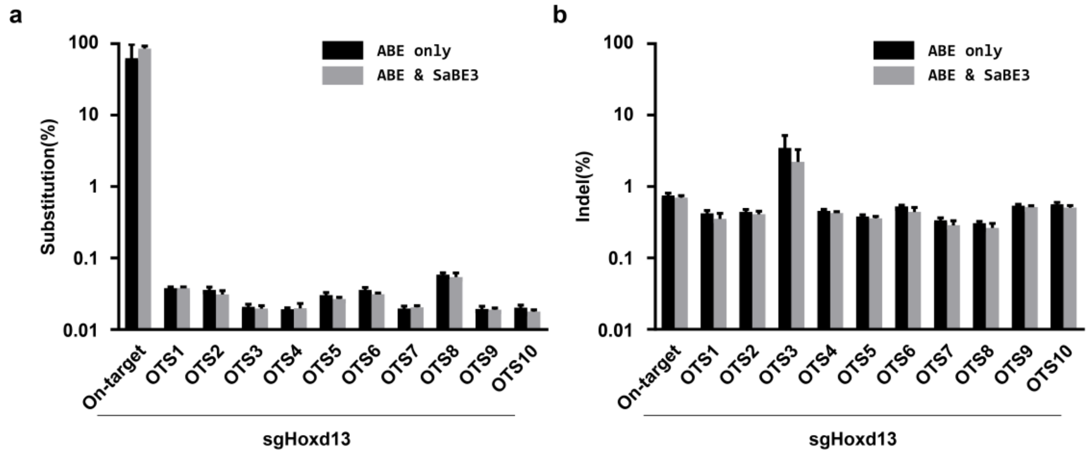

Supplementary Figure 6. Orthogonality analysis for BE and ABE double editing.

(a-b) Targeted deep sequencing was used to measure efficiencies of A-to-G substitution and indel at on-/off-target sites in mutant mice (n=5) and wild type mice (n=5). (a) substitution frequencies and (b) indel frequencies are shown via black and grey columns for ABE only groups and ABE & SaBE3 groups, respectively.

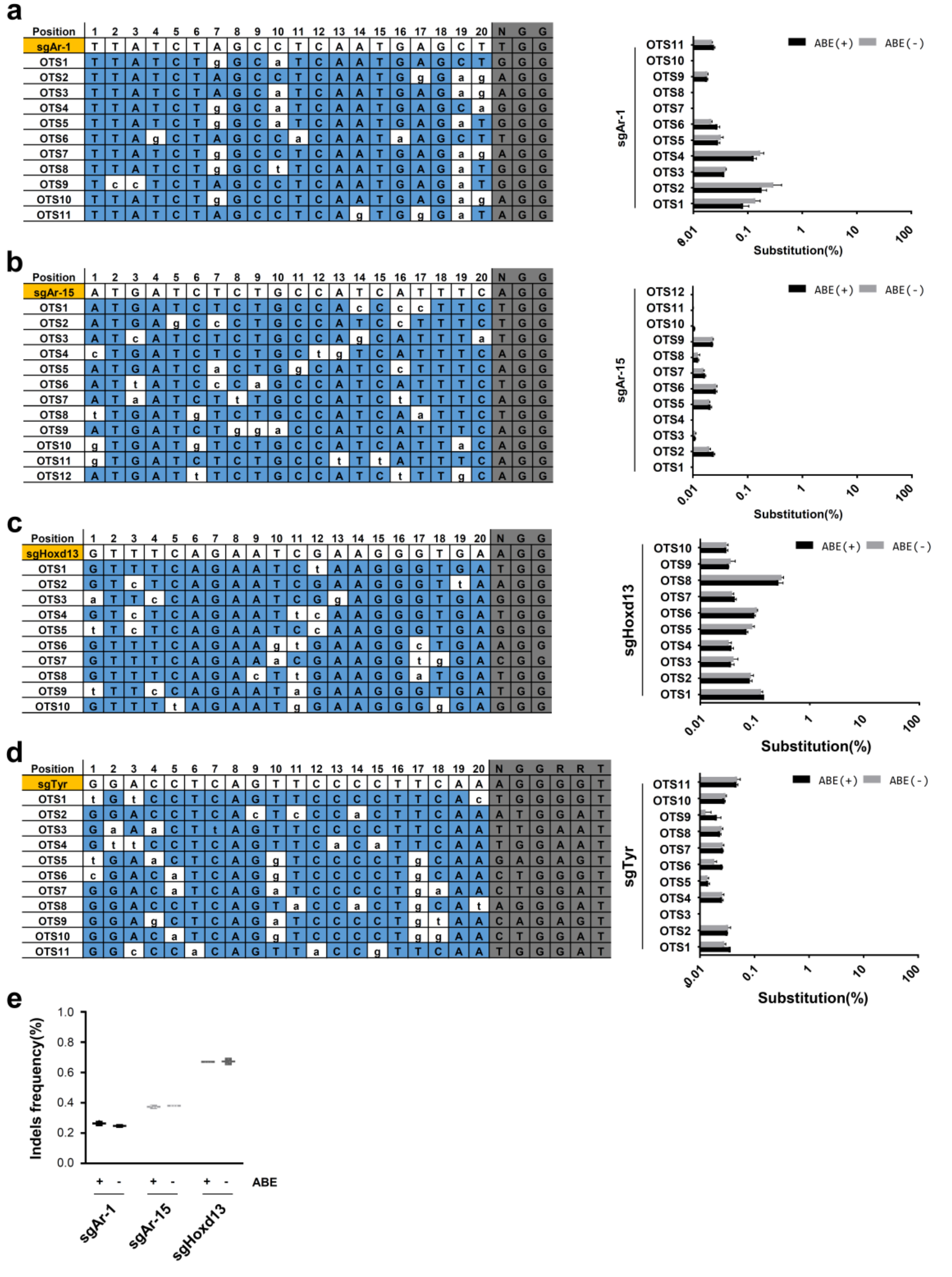

Supplementary Figure 7. The high throughput analysis of off-target and indels by deep sequencing.

(a-d) Targeted deep sequencing was used to measure base editing efficiencies at potential off-target sites in mutant mice (n=3). Left: Mismatched nucleotides and PAM sequences are shown in white and grey boxes, respectively; Right: Substitution frequencies are shown via black and grey columns for ABE(+) groups and ABE(-) groups, respectively. (a) the off-target analysis for sgAr-1; (b) the off-target analysis for sgAr-15; (c) the off-target analysis for sgHoxd13; (d) the off-target analysis

for sgTyr.

(e) Targeted deep sequencing was used to measure indel efficiencies at on-target sites in mutant mice (n=3). +/- indicate ABE groups and control groups.

a

sgHoxd13		Frequency(%)		
WT	GTTTCAGAATCGAAGGGTGAAGG			
H101, ♂	GTTTCGGAATCGAAGGGTGAAGG	7/16	(44)	(Q321R)
H102, ♀	GTTTCGGAATCGAAGGGTGAAGG	6/13	(46)	(Q321R)
H103, ♂	GTTTCGGAATCGAAGGGTGAAGG	6/12	(50)	(Q321R)
H104, ♂	GTTTCGGAATCGAAGGGTGAAGG	5/12	(42)	(Q321R)
H105, ♀	GTTTCGGAATCGAAGGGTGAAGG	9/16	(56)	(Q321R)
H106, ♂	GTTTCGGAATCGAAGGGTGAAGG	8/14	(57)	(Q321R)
H107, ♂	GTTTCGGAATCGAAGGGTGAAGG	9/16	(56)	(Q321R)
H108, ♀	GTTTCGGAATCGAAGGGTGAAGG	11/23	(48)	(Q321R)

Supplementary Figure 8. Germline transmission of the mutant Hoxd13 mouse.

(a) Alignments of modified sequences from F1 newborn pups. The PAM sequences and substitutions are highlighted in blue and red, respectively; N/N indicates positive colonies out of total sequenced. The corresponding targeted codons are shown.

Supplementary Table 1. sgRNAs used in this study

**sgRNA name**	**sgRNA sequence**
**GFP-Scramble**	GACTGGCATCATCTCGCACG
**GFP-sg**	CGTACGTCAGGGTGGTCACGA
**Ar-1**	TTATCTAGCCTCAATGAGCT
**Ar-2**	GCCTCAATGAGCTTGGAGAG
**Ar-3**	AATACTGAATGACCGCCATC
**Ar-4**	GTATTCCTGGATGGGACTGA
**Ar-5**	AAATACCATCAGTCCCATCC
**Ar-6**	AGTGAAGGACCGCCAACCCA
**Ar-7**	CCTTCACTAATGTCAACTCC
**Ar-8**	GGATGTACAGCCAGTGTGTG
**Ar-9**	GCCATCCAAACTCTTGAGAC
**Ar-10**	CTTTCATGCACAGGAATTCC
**Ar-11**	ACTTCGAATGAACTACATCA
**Ar-12**	CTGGTAGAAGCGCCTTGAGC
**Ar-13**	CTGCACAGAATCCAGGAGCT
**Ar-14**	CAAGTCCCATATGGTGAGCG
**Ar-15**	ATGATCTCTGCCATCATTTC
**Ar-16**	CACTGTGTGTGGAAATAGAT
**Ar-17**	TTCCACACACAGTGAAGATT
**Hoxd13**	GTTTCAGAATCGAAGGGTGA
**Tyr**	GGACCTCAGTTCCCCTTCAA

Supplementary Table 2. Primers used for PCR amplification, genotyping and transcription

Primers name	Primer sequence
**GFP-fwd**	TTCCATTTCAGGTGTCGTGG
**GFP-rev**	CAGAGAGAAGTTTGTTGCGCC
**Ar-sg1-T7-fwd**	GCTCGTAAGCTGAAGAAACTTGG
**Ar-sg1-T7-rev**	ACTGTGTGTATCAGTGGATTCAT
**Ar-sg2-T7-fwd**	GCTCGTAAGCTGAAGAAACTTGG
**Ar-sg2-T7-rev**	ACTGTGTGTATCAGTGGATTCAT
**Ar-sg3-T7-fwd**	CAGCCCCACCATTCAGACTT
**Ar-sg3-T7-rev**	TGTTAGGGCCAATGTCAATTTCA
**Ar-sg4-T7-fwd**	CAGCCCCACCATTCAGACTT
**Ar-sg4-T7-rev**	TGTTAGGGCCAATGTCAATTTCA
**Ar-sg5-T7-fwd**	CAGCCCCACCATTCAGACTT
**Ar-sg5-T7-rev**	TGTTAGGGCCAATGTCAATTTCA
**Ar-sg6-T7-fwd**	CAGCCCCACCATTCAGACTT
**Ar-sg6-T7-rev**	TGTTAGGGCCAATGTCAATTTCA
**Ar-sg7-T7-fwd**	CAGCCCCACCATTCAGACTT
**Ar-sg7-T7-rev**	TGTTAGGGCCAATGTCAATTTCA
**Ar-sg8-T7-fwd**	TTTGGGCACAGGGGGAACTA
**Ar-sg8-T7-rev**	ATGGAGAAACTGGTAGAGGCTG
**Ar-sg9-T7-fwd**	TTTGGGCACAGGGGGAACTA
**Ar-sg9-T7-rev**	ATGGAGAAACTGGTAGAGGCTG
**Ar-sg10-T7-fwd**	TTTGGGCACAGGGGGAACTA
**Ar-sg10-T7-rev**	ATGGAGAAACTGGTAGAGGCTG
**Ar-sg11-T7-fwd**	AATTCCCCAGACACACAGACTT
**Ar-sg11-T7-rev**	GACACTGTGACCTGTGTTCCT
**Ar-sg12-T7-fwd**	AATTCCCCAGACACACAGACTT
**Ar-sg12-T7-rev**	GACACTGTGACCTGTGTTCCT
**Ar-sg13-T7-fwd**	AATTCCCCAGACACACAGACTT
**Ar-sg13-T7-rev**	GACACTGTGACCTGTGTTCCT
**Ar-sg14-T7-fwd**	CGGGCAGGATCAAGGATAAAC
**Ar-sg14-T7-rev**	AGCCACAATACGCAGCAGAT
**Ar-sg15-T7-fwd**	CGGGCAGGATCAAGGATAAAC
**Ar-sg15-T7-rev**	AGCCACAATACGCAGCAGAT
**Ar-sg16-T7-fwd**	CGGGCAGGATCAAGGATAAAC
**Ar-sg16-T7-rev**	AGCCACAATACGCAGCAGAT
**Ar-sg17-T7-fwd**	CGGGCAGGATCAAGGATAAAC
**Ar-sg17-T7-rev**	AGCCACAATACGCAGCAGAT
**Hoxd13-sg-T7-fwd**	AGCTTAGGTGTTCCAAGTATCCAGG
**Hoxd13-sg-T7-rev**	TTCACAATGCTTGCCTTTCTAGGC
**Tyr-sg-T7-fwd**	CCAGGGGTTGCTGGAAAAGA
**Tyr-sg-T7-rev**	CATAGGTGCCTGTGGGGATG
**Sry-fwd**	GCACATTTTGGTCAGTGGCT

**Sry-rev**	GCTCTACTCCAGTCTTGCCT
**Gapdh-fwd**	TGAGTGGACCCTTCTTTGTAG
**Gapdh-rev**	CATACCAGGAAATGAGCTTGAC
**IVT-fwd**	TCTCGCGCGTTTCGGTGATGACGG
**IVT-sp-rev**	AAAAAAAGCACCGACTCGGTGCCACTTTTTC
**IVT-sa-rev**	AAAAAAATCTCGCCAACAAGTTG

Supplementary Table 3. Primers used for target deep sequencing

Primer name	Primer sequence
**deep-Tyr-on-F1**	GGGCCCCCAAATCCAAACTT
**deep-Tyr-on-R1**	CCATGGATGGGTGATGGGAG
**deep-Tyr-OST1-F1**	CCCTGCTTCTATGTGGGTGT
**deep-Tyr-OST1-R1**	TCCCAGGGACTAACTAAGCCA
**deep-Tyr-OST2-F1**	GGGGAGCAGAAAGGAATCCA
**deep-Tyr-OST2-R1**	TCAATCCCCTCCCTCATTCCA
**deep-Tyr-OST3-F1**	GGATTAAACCCAGGTCCGCA
**deep-Tyr-OST3-R1**	GAAGAATCATGTCCGCCCCA
**deep-Tyr-OST4-F1**	TCCCAAAACAAAACAAAACTGTGA
**deep-Tyr-OST4-R1**	AACACCTTAGACAGCCGACAT
**deep-Tyr-OST5-F1**	GAGGCTCTGATTTGCCCAGT
**deep-Tyr-OST5-R1**	TCTCACAGAGACCAGAGGTGT
**deep-Tyr-OST6-F1**	TTGCAGCAAAGCTAACCTGG
**deep-Tyr-OST6-R1**	TGTGCTTGCAGAAGATCCCAT
**deep-Tyr-OST7-F1**	CAAGAACCCACGAACAAGCAG
**deep-Tyr-OST7-R1**	CAAGACCAGCACACACAGGG
**deep-Tyr-OST8-F1**	CTTCATGCAGCCACTCCCAC
**deep-Tyr-OST8-R1**	TGCTCTCAAGACCTCTGAGGAAA
**deep-Tyr-OST9-F1**	TGGGCTCCCGTCAAGGTAAT
**deep-Tyr-OST9-R1**	CGTTAAATCTCATGGGCGGC
**deep-Tyr-OST10-F1**	TGTGTGAGATCTGGAGAGGTG
**deep-Tyr-OST10-R1**	CCATTTGCCTGCATGTATGTCT
**deep-Tyr-OST11-F1**	GCAGCAAGGAGCTTGGTTTC
**deep-Tyr-OST11-R1**	GGAGGGCAAGCCTTGAGATT
**deep-Hox-on-F1**	TGCCACGAACCTTTCGGAG
**deep-Hox-on-R1**	AGGATTCACAATGCTTGCCT
**deep-Hox-OST1-F1**	TGAGTCTGCTCTTCTGGATGG
**deep-Hox-OST1-R1**	AAGGACGACTGAAAGTGACCA
**deep-Hox-OST2-F1**	GGTCTGGACACAACTCATTCCA
**deep-Hox-OST2-R1**	CACTTCCACTCTGTCCCTGTC
**deep-Hox-OST3-F1**	TCTAAACACGTCCTCCCCTG
**deep-Hox-OST3-R1**	CCACTACTGGCCTTCCTGTTC
**deep-Hox-OST4-F1**	GCTTGTTGGGAGCACTTAGG
**deep-Hox-OST4-R1**	GGGAAGCTTTGCAGTCCTTTG
**deep-Hox-OST5-F1**	TCCGATGACAGTGCAAGGTT
**deep-Hox-OST5-R1**	ATCGTACGCAGTTTCCTGGG
**deep-Hox-OST6-F1**	TATGGTGGTGTTGTCTGGGTC
**deep-Hox-OST6-R1**	GATGAATCGGCATGTGTGTGC
**deep-Hox-OST7-F1**	TGTGGCAATCAGAGGCATCA
**deep-Hox-OST7-R1**	AGCTGAAGCATGAGAACCCAA
**deep-Hox-OST8-F1**	TCAGTATTGGGGCAGAGGTC

**deep-Hox-OST8-R1**	CCAGCTTGGATCACCACAGA
**deep-Hox-OST9-F1**	CTGAAACCTCAGCCCAGCAG
**deep-Hox-OST9-R1**	ACTGTGACAGGAAGCAGCTTTT
**deep-Hox-OST10-F1**	CCTCTGCGATCCTCTCCTAGA
**deep-Hox-OST10-R1**	TGTGCCTTTCCGGTAGTCAG
**deep-Ar1-on-F1**	GGAAGCCATTGAGCCAGGAG
**deep-Ar1-on-R1**	GCTGCATGAAGTAACGAGTGC
**deep-Ar1-OST1-F1**	ACCCTGCTTCTATGAAGGTGC
**deep-Ar1-OST1-R1**	TCAAACAACCAGAACCCCCAG
**deep-Ar1-OST2-F1**	TTCCCTGGGCATCAAGTCTC
**deep-Ar1-OST2-R1**	GGAAGGAGATGACAACCCCATA
**deep-Ar1-OST3-F1**	AGCACTGATTACCACTTGACCA
**deep-Ar1-OST3-R1**	AGTGCTCTTTCCACTCCTGC
**deep-Ar1-OST4-F1**	CCAGAGCTCCCAGGGACTAA
**deep-Ar1-OST4-R1**	TCCTGGTTTCCCCTCCGTAT
**deep-Ar1-OST5-F1**	AGGGGTTTGCAGTGCCATAA
**deep-Ar1-OST5-R1**	CTCCCCGCTATAGCATTCCC
**deep-Ar1-OST6-F1**	GGCTAGCCTGCTATTATCCG
**deep-Ar1-OST6-R1**	TGGAGTCCATCCCAGGAACA
**deep-Ar1-OST7-F1**	TGTGGCAGATTTTCAGCATGG
**deep-Ar1-OST7-R1**	CCTAAGAGCCCCGCTTTTCT
**deep-Ar1-OST8-F1**	GGAGCTGAAGGGGATTGCAT
**deep-Ar1-OST8-R1**	ATCCCCCTTCCCTGAGACAA
**deep-Ar1-OST9-F1**	ACCCACTGGGACAGAGATGT
**deep-Ar1-OST9-R1**	TGAGCCACCCTTCTGTAGGA
**deep-Ar1-OST10-F1**	ACTCTAACTCTGTTGCCTGCC
**deep-Ar1-OST10-R1**	TTTCCATCCTGATCGATCTCAG
**deep-Ar1-OST11-F1**	TTCCTCTACCCTGGGGCATC
**deep-Ar1-OST11-R1**	GCCAGAAACCATACACTGGC
**deep-Ar15-on-F1**	CAGATTGCAAGAGAGCTGCAT
**deep-Ar15-on-R1**	GGAAAGGGAACAAGGTGGGTT
**deep-Ar15-OST1-F1**	TCCCACTCTTTGGGGCCTAT
**deep-Ar15-OST1-R1**	CCAGCGGGACTTGAAAACTC
**deep-Ar15-OST2-F1**	TCTGCCAGTACCACATCCCT
**deep-Ar15-OST2-R1**	TACTGTCATTGGCCTCGCAG
**deep-Ar15-OST3-F1**	TGGGGATATTCCTCATTTCCTCC
**deep-Ar15-OST3-R1**	CCGGGATCTGTTCTGTCACG
**deep-Ar15-OST4-F1**	TTGGGCTGGGGGAGTTACTA
**deep-Ar15-OST4-R1**	ATCTGTTCGGTTTGCTGGGG
**deep-Ar15-OST5-F1**	GATTCCAGAGCATTCCCCCA
**deep-Ar15-OST5-R1**	AGTCAGCTTGTCTTCACCCC
**deep-Ar15-OST6-F1**	CAGAGTATGGGGAATGGCTCC
**deep-Ar15-OST6-R1**	GGTTGGAGGTCACTACAACACAT

**deep-Ar15-OST7-F1**	TCAGCTGAGCCGCTAACAAA
**deep-Ar15-OST7-R1**	TGGGTTGGATGGAGTTTTGGA
**deep-Ar15-OST8-F1**	CTAGAGAGGGCCCCCATTTTC
**deep-Ar15-OST8-R1**	ACGCCCTGGGCTTGTAAATA
**deep-Ar15-OST9-F1**	TCTGGATTTCTCTGTATCAACTGT
**deep-Ar15-OST9-R1**	CAGGTCTTAGCAGCTTGGGT
**deep-Ar15-OST10-F1**	CCAGACGGCTGACATTTCCT
**deep-Ar15-OST10-R1**	AGCTCACCTCTGTCTCTGTGA
**deep-Ar15-OST11-F1**	ACTCACCTACATAGAGTTAAAGCGG
**deep-Ar15-OST11-R1**	TCTTTGTGTAAAAGCAGTATGGGA
**deep-Ar15-OST12-F1**	TGCCTGGGGTATTGCAATGTT
**deep-Ar15-OST12-R1**	AGCTGTGACATGGGAGGAAAC
